# The variables in the rationing of nursing care in cardiology departments

**DOI:** 10.1186/s12912-023-01222-y

**Published:** 2023-03-03

**Authors:** Barbara Wagner-Łosieczka, Ewelina Kolarczyk, Agnieszka Młynarska, Darek Owczarek, Mikołaj Sadowski, Krystyna Kowalczuk, Beata Guzak, Michał Czapla, Izabella Uchmanowicz

**Affiliations:** 1 Department of Nursing and Obstetrics, Faculty of Health Sciences, Wroclaw Medical University, Wroclaw, Poland; 2 Department of Gerontology and Geriatric Nursing, Faculty of Health Sciences, Medical University of Silesia, Katowice, Poland; 3 Institute of Heart Diseases, University Hospital, Wroclaw, Poland; 4 Department of Integrated Medical Care, Faculty of Health Sciences, Medical University of Bialystok, Bialystok, Poland; 5 Center of Postgraduate Education for Nurses and Midwives, Warsaw, Poland; 6 Department of Emergency Medical Service, Faculty of Health Science, Wroclaw Medical University, Wroclaw, Poland; 7 Group of Research in Care (GRUPAC), Faculty of Nursing, University of La Rioja, Logroño, Spain

**Keywords:** Rationing of nursing care, Burnout, Nurses, Perceived implicit rationing of nursing care (PIRNCA), Maslach Burnout Inventory (MBI), Satisfaction with Life Scale (SWLS)

## Abstract

**Background:**

The rationing of nursing care is a complex process that affects the quality of medical services.

**Purpose:**

An assessment of the impact of nursing care rationing on burnout and life satisfaction in cardiology departments.

**Methods:**

The study included 217 nurses working in the cardiology department. The Perceived Implicit Rationing of Nursing Care, the Maslach Burnout Inventory, and the Satisfaction with Life Scale were used.

**Results:**

A greater emotional exhaustion, the more frequently the rationing of nursing care (r = 0.309, p < 0.061) and the lower the job satisfaction (r=-0.128, p = 0.061). Higher life satisfaction was associated with less frequent rationing of nursing care (r=-0.177, p = 0.01), better quality of care provided (r = 0.285, p < 0.001), and higher job satisfaction (r = 0.348, p < 0.01).

**Conclusion:**

Higher levels of burnout contribute to more frequent rationing of nursing care, poorer evaluation of the quality of care provided, and lower job satisfaction. Life satisfaction is associated with less frequent rationing of care, better evaluation of the quality of care provided, and greater job satisfaction.

## Introduction

According to the definition proposed by Maria Schubert et al., the concept of rationing nursing care (RNC) means withholding or abandoning necessary care for a patient due to a lack of resources. These resources include three basic components: personnel, skills, and time [1]. Meanwhile, the conceptual model of rationing nursing care proposed by Beatrice J. Kalisch assumes that factors such as labor resources, material resources, team activities, and interpersonal communication contribute to the rationing of nursing care [2]. In the absence of one or more of these components, selected nursing activities are omitted or delayed [3]. Currently, the two factors with the most significant impact on the rationing of nursing care are staffing levels and teamwork. When the resources at hand are insufficient to provide the necessary degree of nursing care, a situation arises in which nurses relying on their knowledge are forced to perform only top-priority activities, leaving out those they believe to be “less important” [4]. Undoubtedly, this rationing behavior not only has adverse effects on quality of care and job satisfaction, but also poses the risk of failing to meet the needs of patients, thus presenting a threat to their health or life [5]. With this in mind, poor job satisfaction can lead to burnout, which particularly affects medical professions that are directly involved in interpersonal interactions (human services and helping professions) with patients [6]. A literature review concluded that the rationing of nursing care is associated with a lack of satisfaction with professional work and an increased risk of burnout [7]. The mechanism of burnout was presented by Christina Maslach, who described the process in three dimensions: emotional exhaustion, depersonalization, and reduced sense of efficiency [8]. Emotional exhaustion is expressed as being discouraged from performing professional duties, reduced interest in professional work, constant nervousness, irritability, chronic fatigue, and insomnia. Depersonalization, on the other hand, reflects the objectification of the person being cared for. It involves distancing oneself and treating the recipients of services, including patients, with indifference. The last component of burnout refers to a reduced sense of efficiency in working with the “customer” and a negative perception of professional duties. Constant dissatisfaction, a sense of lacking competency, a lack of self-confidence, and a general sense that superiors are not understanding can be worrisome [9].

Aronson et al. believe that a reduced sense of one’s accomplishments initially preceded by high motivation and high commitment can also lead to burnout [10]. Other authors emphasize the complexity of this process, dividing it into four progressive stages: enthusiasm, stagnation, frustration, and apathy. According to Edelwich and Brodsky, burnout is a symptom of increasing overall disappointment [11]. Gołembiewski and Munzenrider used Maslach’s concept to describe eight phases of burnout. Phase one corresponds to the dimensions of burnout presented by Maslach. Phase two is a high level of depersonalization. The next phases involve interpenetrating high and low levels of each dimension of burnout until the final, eighth phase is reached. In the final phase, a constant and high level of the three dimensions of burnout is observed [12]. Other researchers, namely Demerouti et al., presented a slightly different, simpler model of burnout consisting of two aspects: exhaustion and disengagement. According to the authors, these components develop independently of each other and significantly affect burnout [13].

With the increase in the incidence of cardiovascular diseases, the number of patients requiring treatment and care is increasing. In 2017, the World Health Organization (WHO) announced that cardiovascular diseases are the leading cause of death worldwide every year. More people die of cardiovascular disease than from any other cause [14]. The phenomenon of nursing care rationing is important in assessing the quality of medical services as well as the satisfaction of patients and nurses in cardiology departments [15]. Therefore, undertaking research on the phenomenon of rationing nursing care in a cardiological ward is important in terms of ensuring the quality of care for a patient with cardiac disease [16, 17]. As the number of patients with cardiovascular disease increases, so does the need to ensure quality care for these patients. However, the rationing of nursing care in cardiology wards has not yet been completely investigated. The main objective of our study was to analyze the factors that affect rationing and quality of nursing care in cardiology departments.

## Materials and methods

### Study design and settings

This prospective observational study was conducted in the all cardiology departments in Wroclaw (Poland) from March 2019 to September 2019 using a cross-sectional survey. The nurses were selected through convenience sampling method. All participants were fully informed of the purposes of the study. The inclusion criteria of the study were a status of a registered nurse (RN), at least 12 months of employment at cardiology department in full time and giving consent to participate in the study. The criterion for exclusion from the study was: not giving consent to participate in the study, work on a department other than an cardiology department, work experience less than 1 year, part-time work. Finally The study group consisted of 217 nurses with a mean age of 43.33 years (SD = 9.72) working in the Cardiology Department. The Strengthening the Reporting of Observational Studies in Epidemiology guidelines were followed [18].

### Research instruments

The following standardized survey instruments were used to conduct this study: the Perceived Implicit Rationing of Nursing Care questionnaire (PIRNCA), the Maslach Burnout Inventory General Survey (MBI), and the Satisfaction with Life Scale (SWLS). The PIRNCA questionnaire consists of 31 questions on rationing nursing care and two questions assessing the quality of patient care and the job satisfaction of nurses, which are analyzed separately. In the section devoted to care rationing, the answers to each question were recorded using a four-point scale: never = 0, rarely = 1, sometimes = 2, and often = 3. The final score was an average of the points from questions in which one of the above answers was checked, while questions for which “not applicable” was checked were excluded. Thus, the total score was a number in the range of 0–3 and can be interpreted as follows: a higher score indicates a more implicit rationing of nursing care. The next two questions assessing the quality of patient care and nurses’ job satisfaction used a response scale that ranged from 0 to 10 points, with higher numbers indicating a better quality of patient care and higher job satisfaction, according to nurses [17]. The questionnaire was adapted to Polish conditions; it was shown to be a reliable tool for assessing the level of care rationing and to have a high level of reliability and validity of the translated PIRNCA questionnaire, fully comparable to that of the original [19, 20]. The Cronbach’s alpha was 0.957.

The MBI enables the assessment of burnout using three subscales: emotional exhaustion, depersonalization, and personal accomplishment. Scores on each of these subscales are expressed on a scale of 0–100 points, where a higher score indicates a higher level of burnout. Moreover, total MBI, which is the mean of these three subscales, is also calculated. For questions with yes/no answers, there are no standards to determine whether the level of burnout in respondents is high or low. The MBI questionnaire is a helpful tool used worldwide to determine the effectiveness of burnout reduction measures as part of health policy planning. The MBI questionnaire is an important and reliable survey tool for measuring burnout, well-being and other work-related dimensions [21]. The MBI was developed by Maslach and Jackson in 1981 and consists of three domains: emotional exhaustion (EE), depersonalization (DEP), and reduced personal achievement (PA). A higher score indicates a higher degree of professional burnout. This study used the Polish adaptation of the tool by Pasikowski, which was validated in Polish by Pasikowski and achieved Cronbach’s alpha coefficients of 0.85 for the EE scale, 0.60 for the DEP scale, and 0.76 for the PA scale [22].

The SWLS questionnaire developed by Diener et al. assesses respondents’ sense of life satisfaction. It consists of five statements about the respondent’s life [23]. The SWLS questionnaire was adapted for and translated into Polish by Juczyński [24]. The questions are answered using a 7-point scale with one of the following answers: I completely disagree – 1, I disagree – 2, I slightly disagree – 3, I neither agree nor disagree – 4, I slightly agree – 5, I agree – 6, and I completely agree – 7. Sten scores of 1–4 indicate low life satisfaction, 5–6 indicates average, and 7–10 indicates high life satisfaction [24–26]. Additionally, a sociodemographic questionnaire prepared by the authors was used, which included questions about age, length of service, education, professional specializations, number of jobs, number of patients in care, and marital status. In the Polish validation, the reliability of the SWLS was high (Cronbach’s alpha = 0.891) and all scale questions correlated positively with each other (Pearson coefficient = 0.529–0.797).

### Statistical analysis

The results were systematized and processed quantitatively and qualitatively using a Microsoft Excel spreadsheet. The analysis of quantitative variables using the means (M), standard deviation (SD), medians, quartiles, and minimum and maximum values was performed. The analysis of qualitative variables was carried out by calculating the number and percentage of occurrences of each value. Calculations were performed using the software program R Core Team, version 4.0.2 [27]. The differences between the two groups’ PIRNCA results were tested using the Mann–Whitney test. The comparisons of variables in three or more groups were made using the Kruskal–Wallis test. Once statistically significant effects were detected, post hoc analysis was performed using Dunn’s test to identify statistically significant differences in groups. Correlations between quantitative variables and PIRNCA scores were analyzed using Spearman’s correlation coefficient. Multivariate analysis of the independent effect of multiple variables on the PIRNCA score was performed using linear regression. Qualitative evaluation was carried out by calculating the R2 coefficient. The results were presented in the form of regression model parameters with a 95% confidence interval. Differences with a p-value of < 0.05 were considered statistically significant.

## Results

### Analysis of sociodemographic data

The study included 217 respondents (210 women, 5 men, and no answer given by 2 people). The average age of the respondents was 43.33 years. The youngest person was 21 years old, while the oldest was 60 years old. One hundred and seventy-four people were in a relationship, 40 respondents were single, and 3 people did not answer the question regarding marital status. The vast majority lived in a city/town (N = 180). In terms of the level of education, 108 people had a bachelor’s degree in nursing, 62 had a master’s degree, and 46 respondents had graduated from medical high school. The length of service averaged 21.55 years (SD = 10.53) and ranged from 2 months to 40 years. One hundred and thirty-one participants worked in the Cardiology Department, 57 in the Cardiac Intensive Care Department, and 27 in the Cardiac Surgery Department. One hundred and nine respondents held the title of specialist in a particular field of nursing. The number of patients cared for averaged 16.13 (SD = 12) and ranged from 1 to 48.5. The vast majority of respondents worked between 100 and 200 h per month (187 people) in 12-hour shifts (190 people). The net income ranged from PLN 1,000 to PLN 2,000 for 1 respondent, PLN 2,000 to PLN 3,000 for 27 respondents, PLN 3,000 to PLN 4,000 for 103 respondents, and exceeded PLN 4,000 for 84 respondents; 2 people did not answer the question. The above data is shown in Table 1.

**Table 1:** Characteristics of the participants

Parameter	Group	N	%
Gender	Female Male No answer	210 5 2	96.77% 2.3% 0.92%
Age	20–30 years 31–40 years 41–50 years 51–60 years	30 36 104 47	13.82% 16,59% 47.93% 21.66%
Marital status	Single	40	18.43%
	In a relationship No answer	174 3	80.18% 1.38%
Education	Medical high school	46	21.20%
	Bachelor’s degree	108	49.77%
	Master’s degree No answer	62 1	28.57% 0.46%
Completed specialties	No	108	49.77%
	Yes	109	50.23%
Place of residence	City/Town	180	82.95%
	Country	36	16.59
Form of employment	Cardiology	131	60.37%
	Cardiac Surgery	27	12.44%
	Cardiac Intensive Care	57	26.27%
Length of service	0–10 years 11–20 years 21–30 years 31–40 years No answer	40 48 83 39 7	18.43% 22.12% 38.25% 17.19% 3,23%
Number of working hours per month	Less than 100 100–200 200–300	6 187 23	2.76% 86.18% 10.6%
	300–400	1	0.46%
Shifts	4-hour	1	0.46%
	6-hour 8-hour 12-hour	1 25 190	0.46% 11.52% 87.56%
Net income	1000–2000 PLN	1	0.46%
	2000-3000PLN	27	12.44%
	Over PLN 4,000 No answer	84 2	38.71% 0.92%

* statistically significant relationship (p < 0.05); PLN, polish zloty 1 PLN = 0.2 Euro

### Nursing care rationing assessment (PIRNCA)

The assessments made using the PIRNCA questionnaire had a mean score of 0.97 (SD = 0.56), which indicates that the frequency of rationing care by respondents in cardiology departments “rarely” occurs. The most frequently rationed nursing care activities in cardiology departments include emotional and psychological support for the patient (SD = 1.24), patient education (SD = 1.22), consultation with an external unit (SD = 1.16), and consultation with other members of the interdisciplinary team (SD = 1.14). According to the Mann–Whitney test, there was a statistically significant higher frequency of nursing care rationing among those who were in a relationship compared to those who were single (0.93 ± 0.57 vs. 0.67 ± 0.49; p = 0.006) and those living in rural areas (1.1 ± 0.65 vs. 0.83 ± 0.53, p = 0.029). According to Spearman’s correlation coefficient statistics, the higher the number of patients under one’s care, the more common the rationing of nursing care (r = 0.137; p = 0.046). This data is presented in Table 2.

**Table 2:** Frequency of rationing care in relation to sociodemographic factors

Parameter	Group	Mean ± SD	Median	Quartile	p *
Marital status	Single (N = 40)	0.67 ± 0.49	0.65	0.32–0.9	p = 0.006 *
	In a relationship (N = 174)	0.93 ± 0.57	0.89	0.55–1.21	
Education	Medical high school (N = 46)	0.79 ± 0.48	0.77	0.39–1.06	p = 0.667
	Bachelor’s degree (N = 108)	0.92 ± 0.64	0.77	0.45–1.21	
	Master’s degree (N = 62)	0.87 ± 0.46	0.87	0.58–1.14	
Completed specialties	No (N = 108)	0.89 ± 0.57	0.85	0.45–1.21	p = 0.605
	Yes (N = 109)	0.86 ± 0.56	0.77	0.48–1.14	
Place of residence	City/Town (N = 180)	0.83 ± 0.53	0.77	0.45–1.13	p = 0.029 *
	Country (N = 36)	1.1 ± 0.65	0.91	0.67–1.41	
Form of employment	Cardiology (N = 131)	0.94 ± 0.57	0.9	0.53–1.26	p = 0.068
	Cardiac Surgery (N = 27)	0.78 ± 0.45	0.87	0.52–1.07	
	Cardiac Intensive Care (N = 57)	0.75 ± 0.53	0.68	0.34–1.04	
Number of working hours per month	Less than 200 (N = 193)	0.86 ± 0.57	0.77	0.45–1.15	p = 0.267
	More than 200 (N = 24)	0.99 ± 0.54	0.85	0.63–1.27	
Shifts	12-hour (N = 190)	0.85 ± 0.55	0.77	0.45–1.14	p = 0.089
	8-hour or other (N = 27)	1.07 ± 0.66	1	0.68–1.4	
Net income	Up to PLN 3.000 (N = 28)	0.89 ± 0.69	0.92	0.3–1.23	p = 0.235
	PLN 3.000–4.000 (N = 103)	0.95 ± 0.6	0.9	0.52–1.29	
	Over PLN 4.000 (N = 84)	0.79 ± 0.46	0.74	0.45–1.05	

* statistically significant relationship (p < 0.05); PLN, polish zloty 1 PLN = 0.2 Euro

The analysis of the results from the PIRNCA questionnaire showed that the nurses’ assessment of the quality of patient care was 7.5 points on average (Me = 8 points, Q1 = 6, Q3 = 9, SD = 1.89). Meanwhile, the average job satisfaction score was 6.53 points (Me = 7 points, Q1 = 5, Q3 = 8, SD = 1.94), with typical scores being 5–8 points. The Mann–Whitney test revealed that the quality assessment of patient care was significantly higher among nurses with a specialization (7.82 ± 1.79 vs. 7.19 ± 1.94; p = 0.012). When using the Kruskal–Wallis test in relation to the form of employment, it was found that the quality of patient care was significantly higher (p = 0.001) in the Cardiac Surgery Department (8.42 ± 1.47) and the Cardiac Intensive Care Department (8 ± 1.59) than in the Cardiology Department (7.11 ± 1.98). Detailed data is shown in Table 3.

**Table 3:** Assessment of the quality of patient care

Parameter	Group	Mean ± SD	Median	Quartile	p *
Marital status	Single (N = 40)	7.72 ± 1.77	8	7–9	p = 0.393
	In a relationship (N = 174)	7.43 ± 1.92	8	6–9	
Education	Medical high school (N = 46)	7.67 ± 1.79	8	7–9	p = 0.754
	Bachelor’s degree (N = 108)	7.41 ± 1.94	8	5.75–9	
	Master’s degree (N = 62)	7.52 ± 1.9	8	6–9	
Completed specialties	No (N = 108)	7.19 ± 1.94	7	5–9	p = 0.012 *
	Yes (N = 109)	7.82 ± 1.79	8	7–9	
Place of residence	City/Town (N = 180)	7.55 ± 1.89	8	6–9	p = 0.371
	Country (N = 36)	7.23 ± 1.88	8	5.5–9	
Form of employment	Cardiology (N = 131) — A	7.11 ± 1.98	7	5–9	p = 0.001 *
	Cardiac Surgery (N = 27) — B	8.42 ± 1.47	8.5	7.25–10	
	Cardiac Intensive Care (N = 57) — C	8 ± 1.59	8	7–9	B,C > A
Number of working hours per month	Less than 200 (N = 193)	7.54 ± 1.85	8	6–9	p = 0.476
	More than 200 (N = 24)	7.17 ± 2.21	7	5–9	
Shifts	12-hour (N = 190)	7.56 ± 1.86	8	6–9	p = 0.286
	8-hour or other (N = 27)	7.11 ± 2.1	7	6–8	
Net income	Up to PLN 3.000 (N = 28) — A	7.36 ± 2.18	7.5	5–9	p < 0.001 *
	PLN 3.000– 4.000 (N = 103) — B	6.95 ± 1.98	7	5–8.5	
	Over PLN 4.000 (N = 84) — C	8.2 ± 1.41	8	7–9	C > B

* statistically significant relationship (p < 0.05); PLN, polish zloty 1 PLN = 0.2 Euro

Meanwhile, job satisfaction scores were higher in persons employed in cardiology departments (7.12 ± 1.56 vs. 6.28 ± 1.98, 6.82 ± 1.93; p = 0.048). Furthermore, patient care quality and job satisfaction scores were significantly higher in those with an income exceeding PLN 4,000 (p < 0.001, p = 0.035). Detailed data is presented in Table 4.

**Table 4:** Assessment of job satisfaction

Parameter	Group	Mean ± SD	Median	Quartile	p *
Marital status	Single (N = 40)	6.92 ± 2.13	7.5	5–9	p = 0.115
	In a relationship (N = 174)	6.45 ± 1.89	7	5–8	
Education	Medical high school (N = 46)	7.11 ± 1.69	7	6–8	p = 0.078
	Bachelor’s degree (N = 108)	6.36 ± 2.19	6	5–8	
	Master’s degree (N = 62)	6.36 ± 1.56	7	5–7	
Completed specialties	No (N = 108)	6.34 ± 1.86	6	5–8	p = 0.116
	Yes (N = 109)	6.72 ± 2.01	7	5–8	
Place of residence	City/Town (N = 180)	6.62 ± 1.88	7	5–8	p = 0.136
	Country (N = 36)	6 ± 2.18	6	4–8	
Form of employment	Cardiology (N = 131) — A	6.28 ± 1.98	6	5–8	p = 0.048 *
	Cardiac Surgery (N = 27) — B	7.12 ± 1.56	7	6–8	
	Cardiac Intensive Care (N = 57) — C	6.82 ± 1.93	7	5–8	
Number of working hours per month	Less than 200 (N = 193)	6.51 ± 1.93	7	5–8	p = 0.565
	More than 200 (N = 24)	6.74 ± 2.12	7	5.5–9	
Shifts	12-hour (N = 190)	6.6 ± 1.95	7	5–8	p = 0.154
	8-hour or other (N = 27)	6.07 ± 1.88	6	4–8	
Net income	Up to PLN 3.000 (N = 28) — A	6.57 ± 2.17	7.5	4–8	p = 0.035 *
	PLN 3.000– 4.000 (N = 102) — B	6.2 ± 1.82	6	5–7.75	
	Over PLN 4.000 (N = 83) — C	6.93 ± 1.94	7	5.5–8	C > B

* statistically significant relationship (p < 0.05); PLN, polish zloty 1 PLN = 0.2 Euro

### Burnout assessment (MBI)

The MBI questionnaire showed that the overall burnout score averaged 39.13 points out of 100 possible points and ranged from 0 to 95.83 points (Me = 37.78 points, Q1 = 24.47, Q3 = 52.11, SD = 19.64). The analysis revealed that emotional exhaustion (M = 49.94 points, Me = 50, SD = 32.38) was most responsible for the respondents’ burnout, while depersonalization (M = 30.14 points, Me = 25, SD = 26.95) contributed slightly less and personal accomplishment (M = 30.14 points, Me = 25, SD = 26.95) contributed the least. According to the Spearman correlation coefficient, the greater the emotional exhaustion, the more frequent the rationing of nursing care (r = -0.309; p < 0.061) and the lower the job satisfaction (r = -0.128; p = 0.061). Meanwhile, depersonalization significantly correlated with the rationing of nursing care (r = 0.186; p = 0.007), while personal accomplishment significantly correlated with the quality of nursing care assessment (r = -0.19; p = 0.005).

### Life satisfaction assessment (SWLS)

The results from the SWLS questionnaire were as follows: out of 217 study participants, 98 (45.16%) respondents had a high sense of life satisfaction, 76 (35.02%) had a medium sense of life satisfaction, 40 (18.43%) had a low sense of life satisfaction, and 3 (1.38%) left the questionnaire blank. The analysis of the data using Spearman’s correlation coefficient revealed that the higher the life satisfaction, the less frequent the rationing of nursing care (r = -0.177; p = 0.01), the better the assessment of the quality of care (r = 0.285; p < 0.001), and the higher the job satisfaction (r = 0.348; p < 0.01). The analysis of the data from each scale is presented in Table 5. The correlations between the PIRNCA questionnaire and other scales are shown in Table 6.

**Table 5:** Analysis of the results of individual scales

Parameter		Total (N = 214)
Overall MBI score	mean ± SD	39.13 ± 19.64
	median	37.78
	quartile	24.47–52.11
Emotional exhaustion	mean ± SD	49.84 ± 32.28
	median	50
	quartile	22.22–77.78
Depersonalization	mean ± SD	37.4 ± 28.77
	median	40
	quartile	20–60
Personal accomplishment	mean ± SD	30.14 ± 26.95
	median	25
	quartile	12.5–50
SWLS	Low life satisfaction	40 (18.43%)
	Medium life satisfaction	76 (35.02%)
	High life satisfaction	98 (45.16%)
	No data	3 (1.38%)

MBI — Maslach Burnout Inventory; SWLS — Satisfaction with Life Scale

**Table 6:** PIRNCA correlations with other scales and other quantitative variables

	Care rationing	Assessment of the quality of patient care	Assessment of job satisfaction
**Age**	r = -0.06, p = 0.378	r = 0.104, p = 0.128	r = 0.083, p = 0.224
**Length of service**	r = -0.036, p = 0.607	r = 0.121, p = 0.08	r = 0.075, p = 0.284
**Number of patients in care**	r = 0.137, p = 0.046 *	r = -0.014, p = 0.841	r = -0.08, p = 0.242
**Overall MBI score**	r = 0.189, p = 0.006 *	r = -0.209, p = 0.002 *	r = -0.274, p < 0.001 *
**Emotional exhaustion**	r = 0.141, p = 0.041 *	r = -0.128, p = 0.061	r = -0.309, p < 0.001 *
**Depersonalization**	r = 0.186, p = 0.007 *	r = -0.1, p = 0.146	r = -0.132, p = 0.055
**Personal accomplishment**	r = 0.083, p = 0.23	r = -0.19, p = 0.005 *	r = -0.075, p = 0.277
**SWLS**	r = -0.177, p = 0.01 *	r = 0.285, p < 0.001 *	r = 0.348, p < 0.001 *

r — Spearman’s correlation coefficient

* statistically significant relationship (p < 0.05)

### Impact of each variable on PRINCA

The multivariate linear regression analysis revealed that an independent predictor of the respondents’ assessment of the quality of patient care was working in the Cardiac Surgery Department (parameter = 1.1; 95% = 0.227; CI = 1.972; p = 0.015). Meanwhile, an independent predictor of job satisfaction scores was having a bachelor’s degree (parameter = -0.888; 95% = -1.626; CI = -0.501; p = 0.001) or a master’s degree (parameter = -1.272; 95% = -1.042; CI =- 0.501; p = 0.001) in nursing, as was the overall score on the SWLS questionnaire (parameter = 0.096; 95% = 0.042; CI = 0.15; p = 0.001). Distribution of answers by PRINCA question is presented in Table 7.

**Table 7:** Distribution of answers by PRINCA question (%)

Pytanie	Never		Rarely	Sometimes	Often		No answer		Mean	
**1** Hygiene	34.10%		38.25%	15.67%	3.23%		8.76%		0.87	
**2** Skin care	44.70%		31.80%	12.90%	3.23%		7.37%		0.73	
**3** Bedding	38.25%		34.10%	17.05%	3.69%		6.91%		0.85	
**4** Walking assist	32.26%		34.56%	16.13%	7.37%		9.68%		0.98	
**5** Positions	31.80%		34.10%	21.20%	6.45%		6.45%		1.02	
**6** Bladder or bowel	31.80%		38.25%	17.97%	3.23%		8.76%		0.92	
**7** Food intake	39.17%		32.26%	18.43%	2.76%		7.37%		0.84	
**8** Physical Comfort	38.71%		32.26%	15.67%	4.15%		9.22%		0.84	
**9** Medications	55.30%		23.50%	11.06%	2.76%		7.37%		0.58	
**10** Nutrition	50.23%		24.88%	9.68%	0.46%		14.75%		0.54	
**11** Wound care	54.84%		29.03%	8.76%	1.84%		5.53%		0.55	
**12** Intravenous port	51.61%		28.11%	9.22%	2.76%		8.29%		0.6	
**13** Safe practices	35.02%		37.79%	12.90%	6.45%		7.83%		0.9	
**14** Infections	49.77%		32.26%	10.60%	2.76%		4.61%		0.65	
**15** Education	25.81%		32.72%	24.88%	1.06%		5.53%		1.22	
**16** Preparation	44.24%		35.02%	13.82%	3.23%		3.69%		0.75	
**17** Emotional	20.74%		41.47%	26.27%	8.76%		2.76%		1.24	
**18** Physiological	52.07%		26.73%	13.36%	2.76%		5.07%		0.65	
**19** Behavior	37.79%		31.34%	17.05%	7.37%		6.45%		0.94	
**20** Safety	44.70%		28.57%	17.51%	3.69%		5.53%		0.79	
**21** Missed requests	40.09%		31.80%	17.97%	4.15%		5.99%		0.85	
**22** Waiting time	29.95%		31.34%	25.81%	5.99%		6.91%		1.08	
**23** Member team	23.04%		39.63%	26.27%	5.07%		5.99%		1.14	
**24** External unit	20.74%		36.41%	27.65%	3.69%		11.52%		1.16	
**25** Family member	26.73%		38.25%	29.03%	2.30%		3.69%		1.07	
**26** Delegations	30.88%		41.47%	19.82%	4.15%		3.69%		0.97	
**27** Patient data	29.03%		41.47%	20.74%	4.15%		4.61%		1	
**28** Care plan	41.94%		32.26%	17.05%	2.30%		6.45%		0.78	
**29** Assessment	39.63%		33.64%	17.51%	3.69%		5.53%		0.84	
**30** Nursing process	36.41%		38.71%	18.43%	2.30%		4.15%		0.86	
**31** Nursing plan	38.71%		32.26%	19.82%	3.69%		5.53%		0.88	
**PIRNCA**	**N**	**No answers**	**The range of values**	**Mean**	**SD**	**Median**	**Min**	**Max**	**Q1**	**Q3**
**Assessment of the quality of patient care**	216	1	0–10	7.50	1,89	8	3	10	6	9
**Assessment of job satisfaction**	215	2	0–10	6.53	1.94	7	2	10	5	8

## Discussion

Studies conducted over the past few years have shown that care rationing is widespread around the world, oscillating between 30% and 40% [28, 29]. Human resources, material resources, and communication issues are most often cited as causes of this phenomenon [30]. This study found that the most frequently overlooked areas included emotional and psychological support of the patient, patient education, consultation with other members of the multidisciplinary team, and prompt responses to the needs of the patient. Meanwhile, the literature cites conversing with patients as the most commonly rationed activity [31, 32]. On the other hand, as in this study, a lack of emotional and psychological support for patients and a lack of a prompt response to the needs reported by patients have been confirmed in other studies [33–35]. Perhaps the multitasking that nurses perform forces them to subconsciously select areas where they can afford to ration care more often without much harm to the quality of care and patient safety. A growing body of evidence indicates that nurses are unable to complete all planned care activities and highlights the negative effects this can have on the quality of care and patient outcomes [36].

The analysis found that among sociodemographic factors, the type of education did not affect care rationing. This is confirmed by the results of other authors [31, 32, 36]. Job satisfaction and the quality of nursing care are the two main factors in evaluating the performance of a healthcare system. A study by Janicijevic et al. conducted in Serbia found that patient satisfaction is strongly affected by the satisfaction of the medical staff, while the level of job satisfaction has a significant effect on the quality of care provided [2, 37]. This study demonstrated that both job satisfaction and level of care were rated as average by nurses. Chegini et al. reported that nurses who are satisfied with their cooperation with other team members are less likely to ration care for patients, while nurses who are dissatisfied with their work are 3.4 times more likely to not perform all the tasks required. A previous study unequivocally shows that job dissatisfaction is a significant factor in increased frequency of care rationing, which was also confirmed in this study [36, 38]. The analysis also found that the quality of patient care is an independent factor in determining the level of care rationing, which is in line with the findings of other authors [34, 39].

Our research revealed that among cardiac nurses, burnout results in more frequent rationing of care, especially if the burnout involves emotional exhaustion and depersonalization. In this regard, different research results have been published. For example, Piko et al. found that in Hungary, nurses reported the highest levels of burnout within the emotional domain [40]. Similarly, in a study by Uchmanowicz, emotional exhaustion was the main determinant of burnout [20].

In our study, we found that personal accomplishment significantly affects the quality of care and increases the level of rationing. It is difficult to compare our finding in a group of cardiac nurses because similar studies have not been conducted in Poland or elsewhere. A study by Asgerid et al. noted that nurses derive job satisfaction mainly from positive personal relationships, good working conditions, motivation and recognition, and mutual support within their professional group [41]. According to the questionnaires analyzed in this study, nurses working in cardiology departments showed high satisfaction, which has a direct impact on less frequent rationing of nursing care. This is supported by the study by Uchmanowicz et al., where more pessimistic nurses with low and moderate levels of life satisfaction and nurses with a neutral life orientation had significantly higher nursing rationing scores as measured by the BERCA-R scale than those who were more optimistic and had high levels of life satisfaction [42]. Furthermore, studies by Kalliath and Morris noted that job satisfaction has a significant direct, negative effect on the onset of emotional exhaustion and a significant indirect effect on depersonalization through exhaustion. According to these authors, job satisfaction affects burnout both directly and indirectly, confirming job satisfaction as a significant predictor of burnout [43].

The results of a study by Kalisch et al. conducted in US hospitals showed that job satisfaction among nurses was linked to care rationing. The authors observed that greater job satisfaction correlated with rationing. Although this study did not directly analyze the impact of earnings, the income received for the work performed was an important component [5]. When it comes to net monthly income, it is not possible to directly compare the results of this study with those of other authors. However, it can be argued that the existing literature is consistent with our observation that the level of income affects job satisfaction and the quality of patient care provided.

### Limitations of the study

The present study’s limitations include the relatively small sample of cardiac nurses surveyed (N = 217). However, to date, no research studies have dealt with this topic in cardiac care. Some fact may distort the result e.g. the average value of job satisfaction does not have much predictive power. A worker may be absolutely satisfied with the care provided, while he or she may not be satisfied with the pay. Despite this limitation, the relevance of this study is supported by similar results from various other studies. Therefore, this study is the first step toward learning how to maintain the quality of nursing care in cardiology departments. It is reasonable to plan further studies on larger groups of cardiac nurses and to expand the analysis of rationing care in cardiology departments internationally.

## Conclusion

Burnout is a significant factor in the rationing of nursing care in cardiology departments, the assessment of the quality of patient care, and job satisfaction. The greater the emotional exhaustion, the more common the rationing of nursing care and the lower the job satisfaction. Higher levels of depersonalization result in more frequent rationing of nursing care, while personal accomplishment leads to a poorer evaluation of the nursing care provided. Life satisfaction significantly correlates with nursing care rationing, the assessment of the quality of care provided, and job satisfaction. A high life satisfaction results in less frequent rationing of nursing care, a better assessment of the quality of care provided, and a better evaluation of job satisfaction.

## Data Availability

All relevant data are included with in the manuscript document. If necessary, it is possible to contact the corresponding author to obtain additional materials.

## Abbreviations

M: mean
MBI: Maslach Burnout Inventory
MNC: Missed Nursing Care
PIRNCA: Perceived Implicit Rationing of Nursing Care
SD: standard deviation
SWLS: Satisfaction with Life Scale
WHO: World Health Organization
